# Meta-QTL mapping for wheat thousand kernel weight

**DOI:** 10.3389/fpls.2024.1499055

**Published:** 2024-12-16

**Authors:** Chao Tan, Xiaojiang Guo, Huixue Dong, Maolian Li, Qian Chen, Mengping Cheng, Zhien Pu, Zhongwei Yuan, Jirui Wang

**Affiliations:** State Key Laboratory of Crop Gene Exploration and Utilization in Southwest China, Sichuan Agricultural University, Chengdu, China

**Keywords:** wheat, thousand kernel weight, meta-analysis, QTL mapping, genetic populations

## Abstract

Wheat domestication and subsequent genetic improvement have yielded cultivated species with larger seeds compared to wild ancestors. Increasing thousand kernel weight (TKW) remains a crucial goal in many wheat breeding programs. To identify genomic regions influencing TKW across diverse genetic populations, we performed a comprehensive meta-analysis of quantitative trait loci (MQTL), integrating 993 initial QTL from 120 independent mapping studies over recent decades. We refined 242 loci into 66 MQTL, with an average confidence interval (CI) 3.06 times smaller than that of the original QTL. In these 66 MQTL regions, a total of 4,913 candidate genes related to TKW were identified, involved in ubiquitination, phytohormones, G-proteins, photosynthesis, and microRNAs. Expression analysis of the candidate genes showed that 95 were specific to grain and might potentially affect TKW at different seed development stages. These findings enhance our understanding of the genetic factors associated with TKW in wheat, providing reliable MQTL and potential candidate genes for genetic improvement of this trait.

## Introduction

Wheat breeders are emphasizing trait-based breeding using genotype complementation with elite agronomic traits to accelerate grain yield improvement (Bustos et al., 2013). The identification of quantitative trait loci (QTL) associated with molecular markers is essential for understanding the genetic basis of important traits and is an effective method for improving selection efficiency in breeding programs (Soriano et al., 2021). Breeding for key agronomic and physiological traits related to yield may further enhance the genetic gain of wheat (Tshikunde et al., 2019).

Thousand kernel weight (TKW) is of crucial significance in determining wheat yield, in conjunction with elements like the number of grains per spike and the number of spikes per plant (Avni et al., 2018; Campbell et al., 1999). TKW is predominantly influenced by kernel length (KL), kernel width (KW), kernel thickness (KT), kernel surface area, grain filling rate, and duration time (Guan et al., 2019; Xie et al., 2015; Zanke et al., 2015; Zhai et al., 2018). The inheritance of TKW is relatively stable, exhibiting higher heritability values compared to overall yield, with moderate to high heritabilities ranging from 0.6 to 0.8 (Kuchel et al., 2007). Therefore, the exploitation of genetic variation for TKW and related traits is a promising approach to improve wheat yield (Würschum et al., 2018). The availability of resources, such as draft and complete genome sequences, high-density single nucleotide polymorphism (SNP) arrays and transcriptomic databases has facilitated a powerful approach to identify QTL controlling grain size in wheat, including TKW, KL and KW (Wang et al., 2014a; Winfield et al., 2016; Borrill et al., 2016; Li and Yang, 2017). Numerous QTL/genes for grain size have been identified and characterized using traditional bi-parental linkage mapping and genome-wide association approaches (Cheng et al., 2015; Hu et al., 2015; Krishnappa et al., 2017; Kumari et al., 2018; Qu et al., 2021; Fang et al., 2020; Gao et al., 2021; Mir et al., 2012; Yang et al., 2020), including *TaCKX2* (Zhang et al., 2011), *TaSus* (Jiang et al., 2011), *TaCKX6-D1* (Zhang et al., 2012), *TaGW2* (Yang et al., 2012), *TaGS-D1* (Zhang et al., 2014), *TaGASR7* (Zhang et al., 2015; Dong et al., 2014), *TaCwi* (Jiang et al., 2015), *TaTGW6* (Hu et al., 2016), *TaTGW6-A1* (Hanif et al., 2016), *TaGW2-A1* (Simmonds et al., 2016; Jones et al., 2021), *TaGS5-3A* (Ma et al., 2016), and *TaGL3-5A* (Yang et al., 2019).

However, most of the QTL have minor effects and their expression is highly affected by the environment, the genetic background and their interactions (Zheng et al., 2021). Meta-QTL (MQTL) analysis also allows the identification of putative molecular markers for marker associated selection (MAS) (Soriano et al., 2021; Arriagada et al., 2020; Welcker et al., 2011). Utilizing the MQTL approach has led to significant advancements in integrating different quantitative traits in various crops, such as yield-related traits and insect resistance in maize (Wang et al., 2013; Badji et al., 2018), drought tolerance, and yield-related traits in rice (Khowaja et al., 2009; Raza et al., 2019; Khahani et al., 2020), agronomic and quality traits in cotton (Said et al., 2015). In common wheat, several studies have conducted MQTL analysis for various traits including grain size and shape, grain weight, grain yield, grain protein content, pre-harvest sprouting resistance, adaptation to drought and heat stress, quality traits, tolerance to abiotic and biotic stresses, and resistance against diseases like Fusarium head blight, tan spot, and leaf rust (Gegas et al., 2010; Ma et al., 2022; Liu et al., 2009; Soriano and Royo, 2015; Cai et al., 2019; Tai et al., 2021; Venske et al., 2019; Zheng et al., 2021; Miao et al., 2022; Saini et al., 2022a; Tyagi et al., 2015; Kumar et al., 2020 and Soriano et al., 2017), as well as adaptation to abiotic stresses like drought and heat (Liu et al., 2020a, 2020b). Additionally, phenology, biomass and yield traits MQTL were also identified in durum wheat from 2008 to 2015 (Soriano et al., 2017).

This study is aimed at identifying genetic factors from diverse genetic populations with the potential to enhance TKW in wheat. To compare the differences in TKW QTL among diverse genetic populations, we gathered 28 double haploid (DH) populations, 16 F_2_ populations, and 76 recombinant inbred line (RIL) populations across multiple environmental conditions. By conducting MQTL analysis using publicly available reference data, we obtained 66 MQTL and subsequently identified 96 candidate genes within these MQTL regions that might influence TKW.

## Materials and methods

### Collection of QTL for TKW and construction of reference map

For QTL controlling for TKW, a comprehensive collection was performed using PubMed (http://www.ncbi.nlm.nih.gov/pubmed), Google Scholar (https://scholar.google.com/) and China National Knowledge Infrastructure (https://www.cnki.net/). For each initial QTL, the necessary information was collected: (i) QTL name, (ii) thousand kernel weight trait, (iii) flanking or closely linked marker, (iv) position of QTL (peak position and/or confidence intervals), (v) type and size of lines in the mapping population (F_2_, DH, RIL and Backcross), (vi) LOD (logarithm of the odds) value for each QTL, and (vii) percentage of phenotypic variance explained for each QTL (PVE or R^2^). For some QTL for which the LOD and R^2^ values were missing in the previous studies, they were respectively assumed to be 3 and 10% as the common practice (Khahani et al., 2020; Venske et al., 2019). When the peak position was missing, the midpoint between the two flanking markers was treated as the peak position (Yang et al., 2021). In addition, for the initial QTL which were missing flanking markers and confidence intervals (CIs), the CIs were recalculated according to the population type and size using the following standard formula: (i) F_2_ and backcross population, CI=530/(N×R^2^), (ii) recombinant inbred line (RIL) population, CI=163/(N×R^2^), and (iii) doubled haploid population, CI=287/(N×R^2^). Here, 530, 163, and 287 are the population-specific constants obtained from different simulations (Darvasi and Seller, 1997; Guo et al., 2006). Where N is the size of the mapping population used for QTL analysis, and R^2^ is the phenotypic variation explained by QTL (Kumar et al., 2020). The primary markers, including Simple Sequence Repeats (SSR), Diversity Arrays Technology (DArT), and the 9K/55K/90K/660K iSELECT SNP markers, have been utilized to construct genetic linkage maps for QTL mapping studies, as reported in a previous research (Liu et al., 2020).

### Construction of consensus genetic maps

The genetic maps, comprising multiple markers extensively utilized in various QTL mapping studies, were employed in the construction of a reference genetic map. (i) “Wheat, Consensus SSR, 2004” and “Wheat, Composite, 2004” (consisting of 4403 SSR, RFLP, and AFLP markers), as well as “Wheat, Synthetic ×Opata, BARC”, were all obtained from the GrainGenes website (https://wheat.pw.usda.gov/GG3/), (ii) “Wheat consensus map version 4.0” downloaded from the website (https://www.diversityarrays.com), (iii) A SSR consensus map (1235 SSR markers) (Somers et al., 2004), (iv) A consensus map (including 3669 DarT & SSR-integrated map) for durum wheat (Marone et al., 2013), (v) Three SNP genetic maps, namely those derived from the 9 K iSelect Beadchip Assay (3959 Illumina 9 K iSelect Beadchip Array), iSelect 55 K SNP Assay, and iSelect 90 K SNP Assay (40268 Illumina iSelect 90 K SNP Array) based on the Illumina platform, and genotyping by sequencing (GBS) (Cavanagh et al., 2013; Saintenac et al., 2013; Venske et al., 2019; Wang et al., 2014b). The R package LR merge was utilized to construct the reference map for this Meta-QTL study using the optimized “synthetic” method, which enables the generation of genetic maps across multiple populations, as described by Venske et al. (2019), (vi) A consensus map (AxiomR Wheat 660 K SNP array) which was made by Cui et al., 2017, (vii) A high-density consensus map, which integrates 14548 SSR, DarT, 90 K, and 660K SNP markers sourced from two dense genetic maps (Maccaferri et al., 2015; Soriano and Alvaro, 2019), was established and served as a reference map (Bilgrami et al., 2020). This comprehensive map spans a total length of 4813.72 cM, covering the 21 linkage groups ranging from 155.6 cM and 350.11 cM. The reference map was used to project individual QTL identified in separate populations (Shariatipour et al., 2021), and it served as the reference map for our research.

### Projection of QTL and meta-QTL analysis

The initial QTL data, individual genetic maps from previous independent studies, and reference genetic maps were utilized as input files to construct a consensus map. Subsequently, MQTL analysis was carried out as described by Yang et al. (2021) (Yang et al., 2021). The projection was conducted using BioMercator v4.2 software (Sosnowski et al., 2012; Arcade et al., 2004). The initial QTL and the details of each QTL, for example, CI, the peak position, LOD score and R^2^, were projected onto a reference map (Arcade et al., 2004). QTL were discarded when they could not be projected onto the consensus map or when they mapped to positions outside the consensus map (Kumar et al., 2020).

After projection, MQTL analysis was performed on each chromosome using BioMercator v4.2 software via the Veyrieras two-step algorithm (Sosnowski et al., 2012; Arcade et al., 2004; Goffinet and Gerber, 2000). Two different approaches were used based on the number of initial QTL on each chromosome. In the first approach, when the number of QTL per chromosome was 10 or fewer, the approach of Goffinet and Gerber (2000) was carried out (Sosnowski et al., 2012). Based on this approach, the best MQTL model with the lowest AIC values for QTL integration and identification of consensus MQTL positions in BioMercator v4.2 software was selected. However, if the number of QTL in a chromosome was more than 10, the second method proposed by Veyrieras was used (Veyrieras et al., 2007). In accordance with this approach, meta-analyses were conducted for individual chromosomes by using a two-stage approach available in the software. In the first step, the collected QTL on individual chromosomes are clustered using default parameters. The number of potential MQTL per chromosome is then estimated based on the following five selection criteria, including AIC, AICc, AIC3, BIC and AWE (AIC = Akaike information criterion, AICc = corrected Akaikes information criterion, AIC3 = A variant of AIC that uses 3p as the penalty term, BIC = Bayesian information criterion, and AWE =approximate weight of evidence).

A QTL model which had the lowest values of the selection criteria was regarded as the best optimal model for the next step of meta-analysis. In the second step, the 95% CI and the positions of each MQTL was determined in accordance with the optimal model selected in the previous step. The QTL were integrated in such a way that the peak position of the initial QTL fell within the MQTL CI (Daryani et al., 2022), and MQTL with the minimum AIC values were retained for further analysis.

### Identification of putative genes in MQTL regions

All identified MQTL were subsequently aligned to the wheat reference genome. The markers located on either side of the MQTL confidence interval were manually searched. Their respective flanking or primer sequences were derived from Triticeae Multi-omics Center (http://wheatomics.sdau.edu.cn), annotated by IWGSC_v1.1_HC_gene. They were also obtained from resources like the Illumina company website (https://www.illumina.com), URGI Wheat (http://wheat-urgi.versailles.inra.fr), GrainGenes (https://wheat.pw.usda.gov/GG3/), and DArT (https://www.diversityarrays.com). The putative genes are located within the regions identified based on the positions of the flanking markers of the MQTL (or the marker closest to the flanking markers) (Kumar et al., 2020). The sequence information was then aligned to the wheat reference genome in the Triticeae Multi-omics Center (http://wheatomics.sdau.edu.cn). This was done by using the BLASTN program to find the physical position of flanking markers (Yang et al., 2021). In addition, the physical locations of some SSR, SNP and DArT markers provided in the previous researches were also utilized as reference (Wang et al., 2014b; Cabral et al., 2018).

Three methods have been used to identify putative genes within MQTL regions (Venske et al., 2019; Yang et al., 2021). (i) In the first method, given the leading position of rice in gene function study, the strategy of wheat-rice orthologous comparison was employed to mine the key candidate genes in the MQTL region. For this purpose, the China Rice Data Center (https://www.ricedata.cn/gene/) was manually utilized to identify the genes for TKW associated traits in rice. In addition, the homologous genes of wheat were retrieved from the Triticease-Gene Tribe (http://wheat.cau.edu.cn/TGT/) based on the IWGSC RefSeq v1.1. The genes located in the MQTL region were regarded as important candidate genes influencing wheat yield and yield-related traits. (ii) To further refine the MQTL, those having at least two overlapping initial QTL with a physical distance of less than 20.0 Mb and a genetic distance of less than 1.0 cM, which were referred to as core MQTL, were selected in the second approach. (iii) The peak physical positions of the remaining MQTL were calculated using 1-Mb region on each side of the MQTL to identify relevant genes within the MQTL regions. The peak physical position of the MQTL was calculated according to the method proposed by Saini (Saini et al., 2022b). Both the original and estimated ranges of physical positions were then input into the search toolbox of the “Gene” in the WheatGmap database to obtain details of gene models (locus ID information and functional descriptions) corresponding to MQTL regions (Zhang et al., 2021).

### Expression of candidate genes within MQTL regions

Gene expression analysis examines how genes are transcribed to produce functional products such as RNA or proteins (Gudi et al., 2022). The GENEDENOVO cloud platform (https://www.omicshare.com/tools) was used to perform the GO and KEGG analysis. For transcriptional expression analysis, the Expression Visualization and Integration Platform (expVIP, http://www.wheat-expression.com) with expression data from spike and seed stages was employed in this study (Borrill et al., 2016; Ramírez-González et al., 2018). Only candidate genes showing at least 2 TPM of expression were considered (Wagner et al., 2013). The expression characteristics of candidate genes were displayed by the heat map of TPM using the TBtools software (Chen et al., 2020).

## Results

### Collection of QTL controlling TKW in wheat

**Figure 1:**
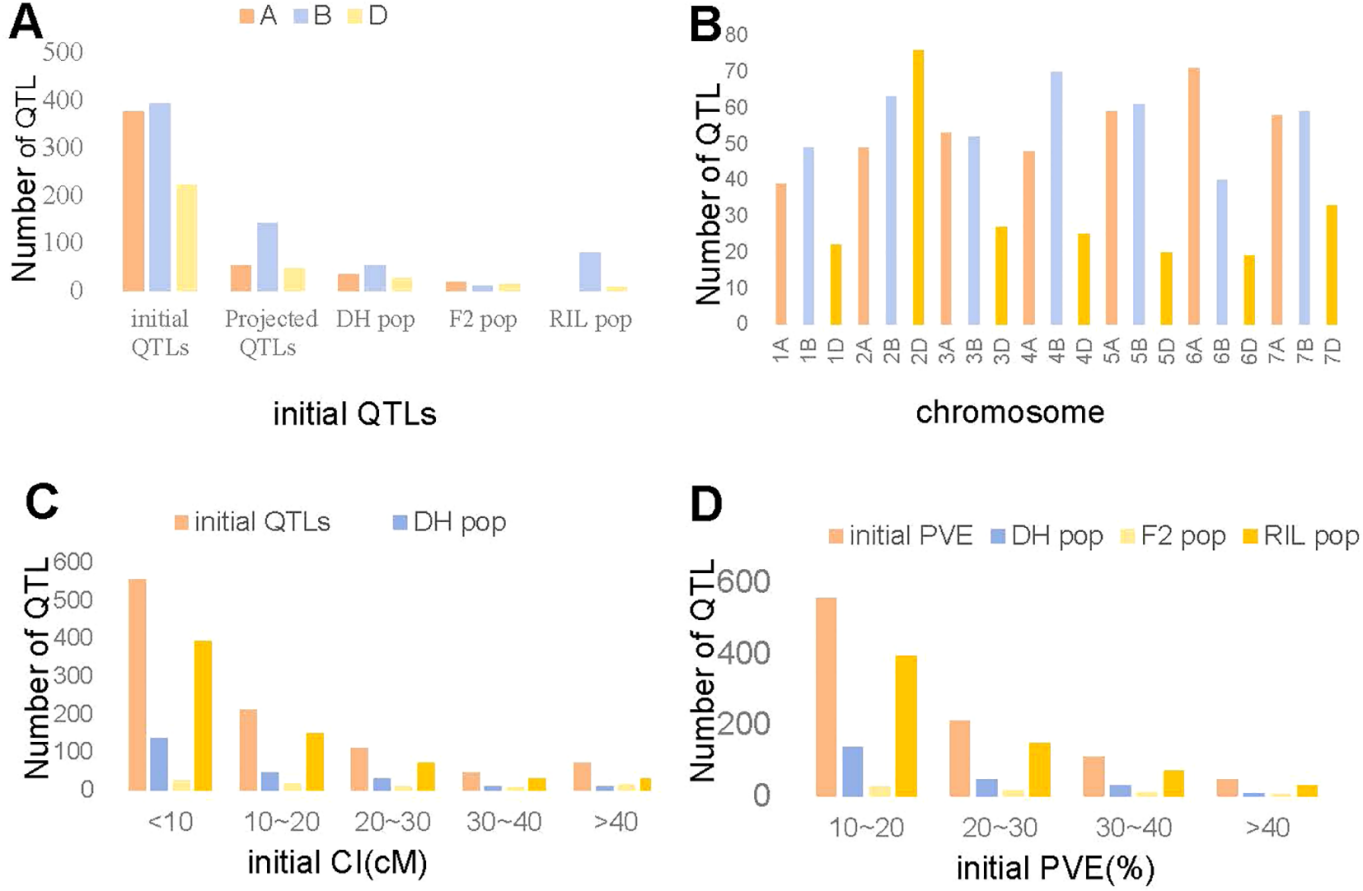
Analysis of collected 993 QTL. **(A)** Number of initial and projected QTL in the DH, F_2_ and RIL population. **(B)** Number of QTL on each chromosome. **(C)** Confidence intervals of the initial QTL in the DH, F_2_ and RIL population. **(D)**. Individual PVE of QTL in the DH, F_2_ and RIL population.

We undertook an extensive review of 120 studies published between 2008 and 2023, which encompassed 28 double haploid (DH) populations, 16 F_2_ population, and 76 RIL populations to collect data on available QTL (**Supplementary Table S1**). A total of 233, 81, and 679 initial QTL associated with TKW were identified and distributed across all 21 wheat chromosomes in the DH, F_2_ and RIL populations, respectively. Among the previously identified 993 initial QTL, 37.97% were allocated to subgenome A, 39.68% to subgenome B, and only 22.36% to subgenome D (**Figure 1A**). Only 242 QTL were successfully projected onto the consensus map, with 114, 42, and 86 QTL for the DH, F_2_ and RIL populations, respectively (**Figure 1A**). The markers related to the remaining 119, 39, and 593 QTL were either absent in the consensus map or were characterized by low phenotypic variation explained (PVE) values or large CI (**Figure 1A**). Subgenome B had the largest count of 142 QTL, while subgenome D had the smallest with only 47 QTL (**Figure 1A**). In general, the number of QTL per chromosome ranged from 19 on chromosome 6D to 76 on chromosomes 2D, with an average of 47 QTL per chromosome (**Figure 1B**). The CI of these QTL ranged from 0.02 cM to 56.72 cM, approximately 55.89% had a CI less than 10 cM and 78.45% had a CI less than 20 cM (**Figure 1C**). The PVE values for individual QTL ranged from 0.7% to 54.0%, with an average of 9.91% (**Figure 1D**). Only 33.13% of the initial QTL showed PVE values greater than 10% (**Figure 1D**).

**Figure 2:**
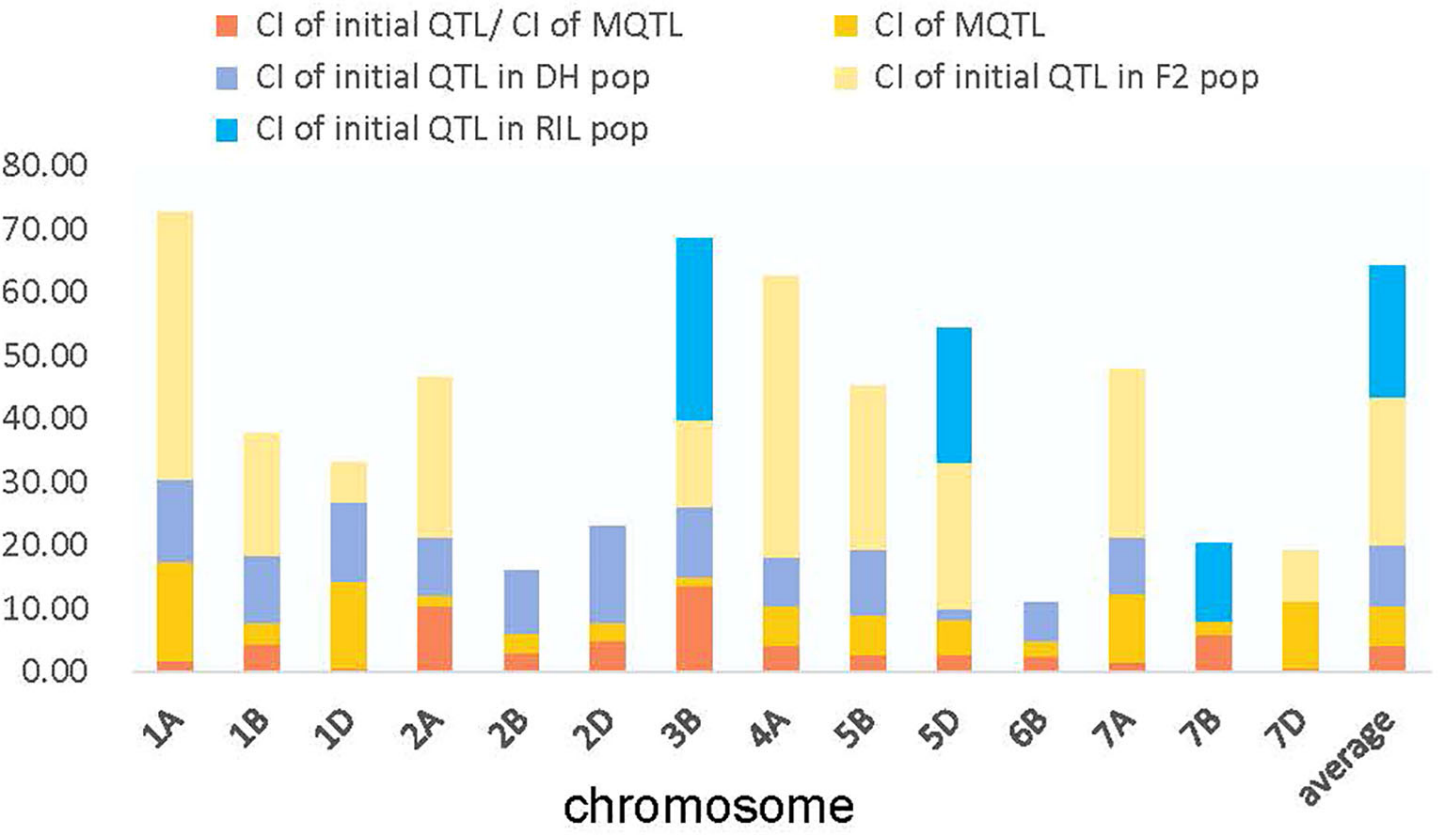
Comparison of mean CI for initial QTL and MQTL.

**Figure 3:**
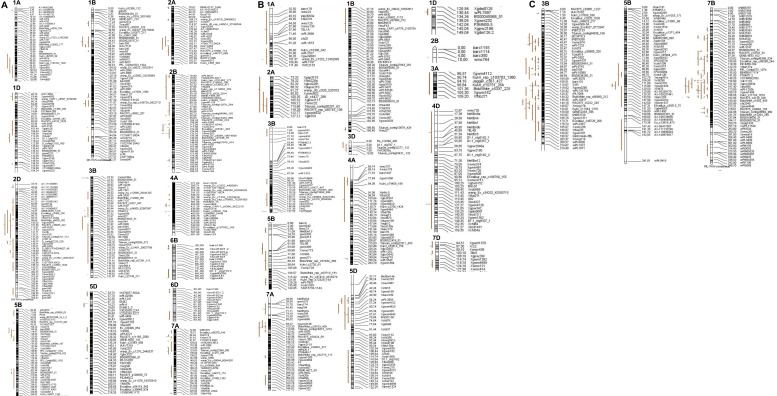
Distribution of the MQTL on chromosomes. **(A)** On the chromosome 1A, 1B, 1D, 2A, 2B, 2D, 3B, 4A, 5B, 5D, 6B, 6D, and 7A in the DH population. **(B)** On the chromosome 1A, 1B, 1D, 2A, 2B, 3A, 3B, 3D, 4A, 4D, 5B, 5D, 7A, and 7D in the F2 population. **(C)** On the chromosome 3B, 5B, and 7B in the RILpopulation. Original TKW QTLs were detected and are represented by red bars. Black bars within the chromosomes indicate marker density, and to the right of these bars is the distance in cM along with the marker names.

**Figure 4:**
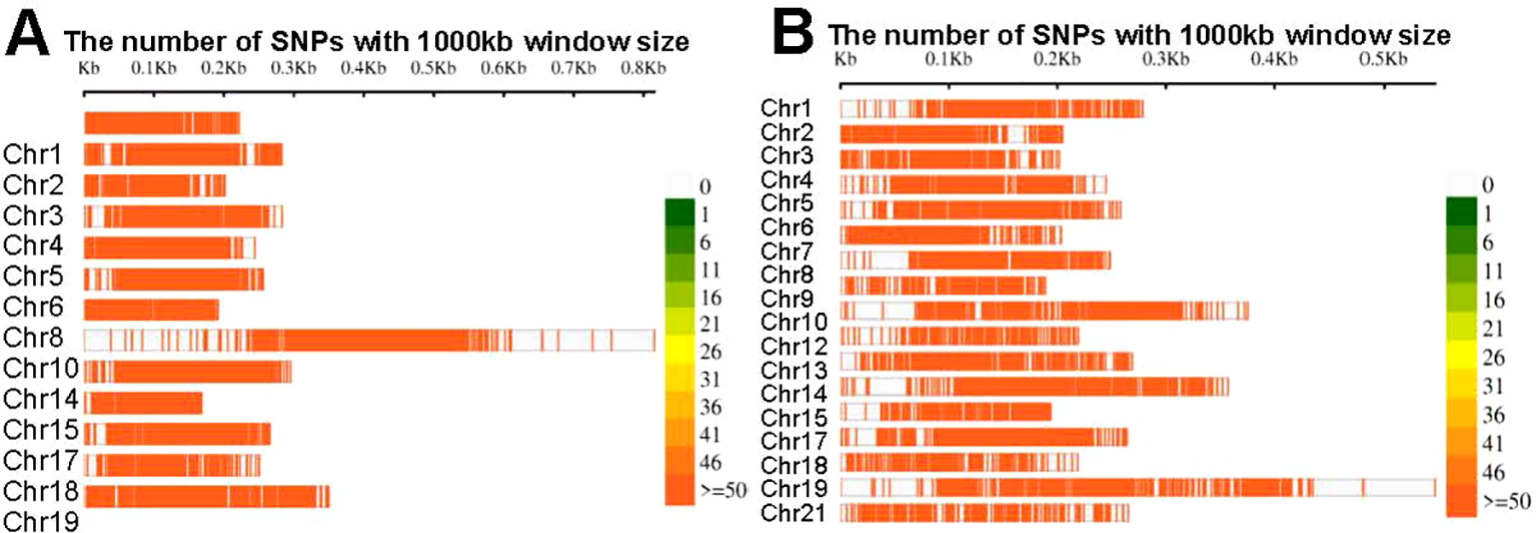
SNPs density in the **(A)**. DH populations and **(B)** F_2_ populations.

The 86 QTL that were projected were identified on chromosomes 3B, 5D, and 7B in the RIL population (**Figures 2**, **3**). The 114 QTL projected were identified on 13 wheat chromosomes, excluding 3A, 3D, 4B, 4D, 5A, 6A, 7B, and 7D in the DH population (**Figures 2**–**4**). The 42 QTL projected were identified on 14 wheat chromosomes, excluding 2D, 4B, 5A, 6A, 6B, 6D, and 7B in the F_2_ population (**Figures 2**–**4**).

### Construction of a high−density consensus genetic map

The reference genetic map, which encompassed SSR, DArT, SNP markers, and a few genes, was employed for subsequent Meta-QTL analysis (**Supplementary Table S2**). Subsequently, 28 DH populations, 16 F_2_ populations, and 76 individual genetic maps of RIL were aligned onto the reference map. Ultimately, three high-quality consensus genetic maps were constructed, comprising 16849, 11999, and 5380 markers, with total lengths of 3828.59 cM, 4537.87 cM, and 858.99 cM, respectively (**Supplementary Tables S3**–**S5**). The average length of each chromosome was 294.51 cM, 266.93 cM, and 286.33 cM in DH, F_2_ and RIL populations, respectively (**Supplementary Tables S3**–**S5**), which was consistent with the previous study (Venske et al., 2019). These markers were distributed unevenly on chromosomes, with chromosome 4A/7A/7B containing the most markers (1471/980/2693), forming the longest linkage groups of 815.67 cM, 546.97 cM, and 348.7 cM in DH, F_2,_ and RIL populations, respectively (**Supplementary Tables S3**–**S5**).

The marker density at the fore-end of the chromosome was significantly higher than that at the end (**Figure 4**). This difference was primarily due to the diverse numbers and types of markers present in the independent genetic maps used for constructing the consensus map. Overall, this consensus map was constructed using a substantial amount of marker information.

### Projection of initial QTL and identification of meta−QTL for TKW

To further narrow down the CI of causal genes, a meta-analysis was performed using information such as the lowest model value and a minimum of two overlapping initial QTL from the 242 projected QTL. As a result, 66 meta-QTL (MQTL) distributed on all 21 wheat chromosomes were obtained **(Supplementary Table S6**). The 95% CI of these MQTL, with an average of 5.41 cM, exhibited a 3.06-fold reduction compared to the initial QTL (**Figure 2**; **Supplementary Table S6**). More significantly, both genetic and physical locations of these MQTL-corresponding markers were provided through a consensus map (**Supplementary Table S6**). The physical locations of the 66 MQTL, determined by flanking marker sequences, ranged from 0.13 Mb (*MQTL.DH.7A.1*) to 52.66 Mb (*MQTL.RIL.5D.2*) (**Supplementary Table S6**). These MQTL were then selected for further analysis to identify potential candidate genes. Among the identified MQTL, 12 were classified as core MQTL, meeting the criteria for candidate gene search in available databases (**Table 1**). The physical distances of the core MQTL ranged from 0.49 to 14.12 Mb, while their genetic distance ranged from 0.02 to 0.85 cM (**Table 1**; **Supplementary Table S6**).

**Table 1:** Depiction of 12 core MQTL identified for thousand kernel weight in wheat.

MQTL ID	Initial QTL	Physical interval(bp)	Interval distance (Mb)	Chr	Position (cM)	CI (cM)	Genetic interval (cM)	Left marker	Right marker
*MQTL.DH.1B.3*	2	667717050-670783640	3.06	1B	228.14	0.2	228.04 - 228.24	Tdurum_contig61914_169	BS00000010_51
*MQTL.DH.2A.1*	2	36632177-36138748	0.49	2A	98.23	0.76	97.85 - 98.61	Ra_c2798_261	BobWhite_c26374_339
*MQTL.DH.2A.3*	4	663328967-675938678	12.61	2A	159.43	0.29	159.28 - 159.57	BS00022241_51	GENE-1792_762
*MQTL.DH.3B.2*	2	605269005-602356133	2.91	3B	69.04	0.59	68.74 - 69.33	BobWhite_c12281_752	Tdurum_contig44013_164
*MQTL.DH.5B.2*	4	457342612-460678316	3.34	5B	209.76	0.02	209.75-209.77	wsnp_Ku_c17875_27051169	Kukri_c10530_1013
*MQTL.DH.7A.3*	3	719567282-722303439	2.73	7A	125.37	0.61	125.065-125.68	Kukri_c9728_1171	RFL_Contig2834_890
*MQTL.RIL.3B.2*	2	736712583-737764228	1.05	3B	83.83	0.85	83.41-84.26	IAAV6566	wsnp_Ex_c3907_7088011
*MQTL.RIL.3B.5*	4	81818278-95150976	13.33	3B	105.36	0.83	104.95-105.78	wsnp_Ex_c21930_31102213	IAAV7128
*MQTL.RIL.3B.7*	2	820501695-818275897	2.22	3B	156.45	0.6	156.15-156.75	RAC875_c37741_476	BobWhite_c45118_495
*MQTL.RIL.3B.8*	3	769345579-783472149	14.12	3B	159.88	0.13	159.82-159.95	GENE-0293_346	wsnp_Ra_c7158_12394405
*MQTL.RIL.7B.3*	3	84215789-91292649	7.07	7B	151.55	0.4	151.35-151.75	IAAV3391	IACX17
*MQTL.RIL.7B.7*	4	726970893-730164735	3.19	7B	240.56	0.29	240.42-240.71	wPt-6484	wPt-7046

MQTL, Meta-QTL; CI, the confidence interval; Chr, chromosome.

### Putative genes and their expression analysis

We employed three approaches to identify potential candidate genes related to TKW in wheat. An extensive search for known rice genes associated with TKW traits resulted in 106 rice known genes (**Supplementary Table S7**), which were then used to identify their wheat homologs. Among these, only 42 genes were located within 36 MQTL regions (**Supplementary Table S8**). The development of grain weight is influenced by various molecular and genetic factors that lead to dynamic alterations in cell division, expansion, and differentiation.

Within each MQTL, the number of potential genes ranged from one to eight, with an average of 2.82 genes per MQTL being homologous to rice (**Supplementary Table S8**). A total of 4913 genes were identified within the MQTL regions, encompassing the 42 genes with corresponding rice homologs for TKW, and an additional 4844 putative genes after eliminating duplicates in overlapping MQTL (**Supplementary Table S9**). The majority of these potential genes (393 genes) were located within the confidence region of *MQTL.RIL.5D.2* (**Supplementary Table S6**). In contrast, only one gene was found in *MQTL.DH.1B.3*, *MQTL.DH.2B.2*, *MQTL.DH.2B.3*, *MQTL.DH.5B.1*, *MQTL.DH.5D*, and *MQTL.DH.7A.1* etc being homologous to rice (**Supplementary Tables S6**, **S8**). These identified genes with similar functions included a diverse assortment of protein families and domains, including 390 for protein kinases, 153 putative genes for F-box-like domain proteins, 73 for Glycosyltransferase family proteins, 67 for cytochrome P450 proteins, 46 for leucine-rich repeat domain proteins, 45 for Pentatricopeptide repeat-containing protein, and 44 for BTB/POZ domain-containing proteins, etc (**Supplementary Table S9**).

**Figure 5:**
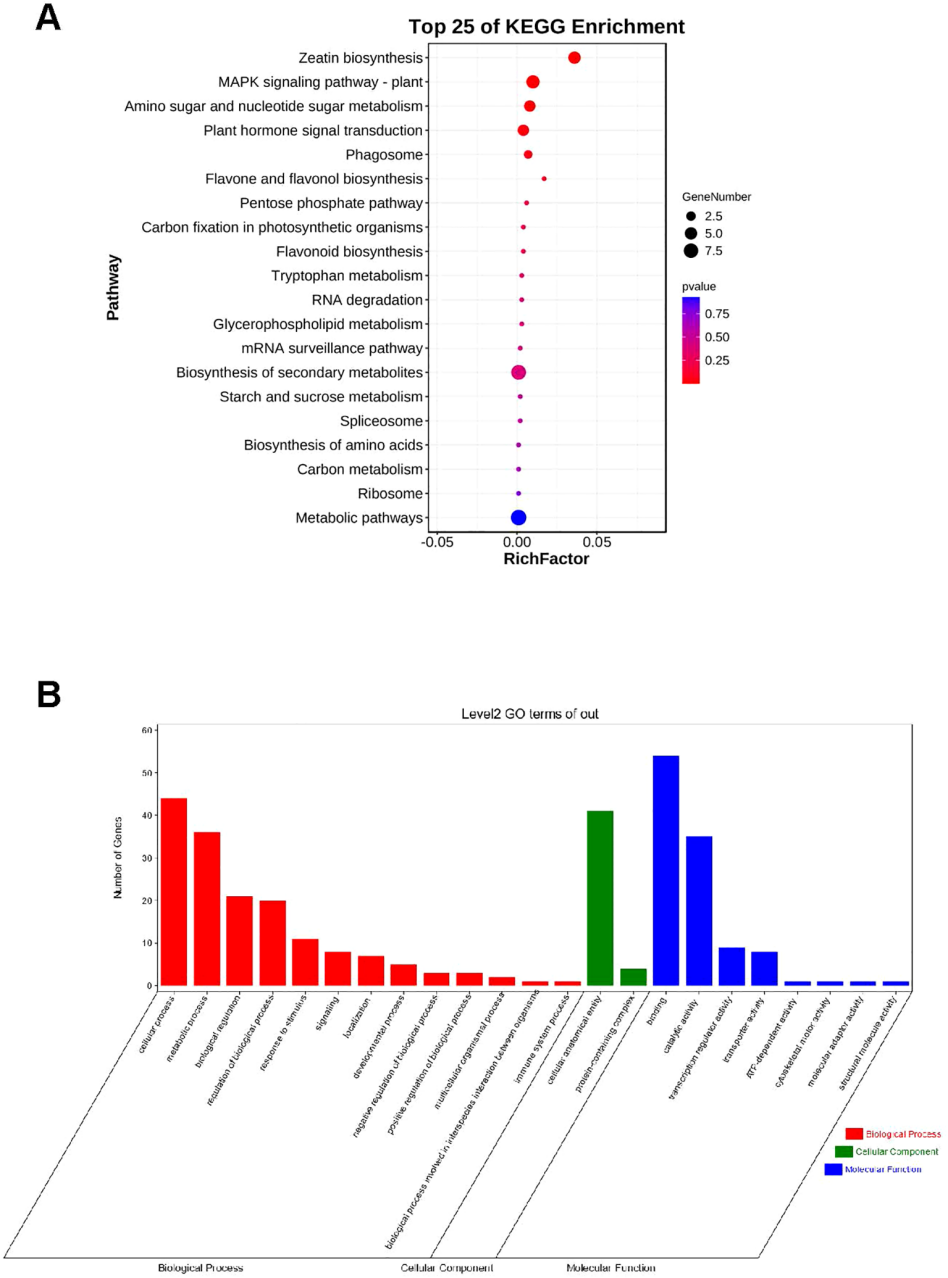
**(A)** KEGG pathway enrichment of 95 candidate genes. **(B)** GO terms for 95 candidate genes underlying MQTL interval for thousand kernel weight.

To ascertain the functional classification of the identified 95 genes (**Supplementary Table S8**), we conducted Gene Ontology (GO) enrichment and Kyoto Encyclopedia of Genes and Genomes (KEGG) pathway analyses. The KEGG enrichment analysis indicated that these potential genes play significant roles in Zeatin biosynthesis, the MAPK signaling pathway in plants, amino sugar and nucleotide sugar metabolism, and plant hormone signal transduction, with Metabolic pathways having the largest number of putative genes (**Figure 5A**). The most enriched GO terms in biological processes were associated with metabolic processes and cellular processes (**Figure 5B**). The most enriched GO terms in molecular functions were involved in binding and catalytic activities (**Figure 5B**). Concerning cellular components, genes were mainly enriched in cellular anatomical entities and protein-containing complexes (**Figure 5B**). We identified the 95 critical genes that regulate TKW for subsequent gene expression analysis.

**Figure 6:**
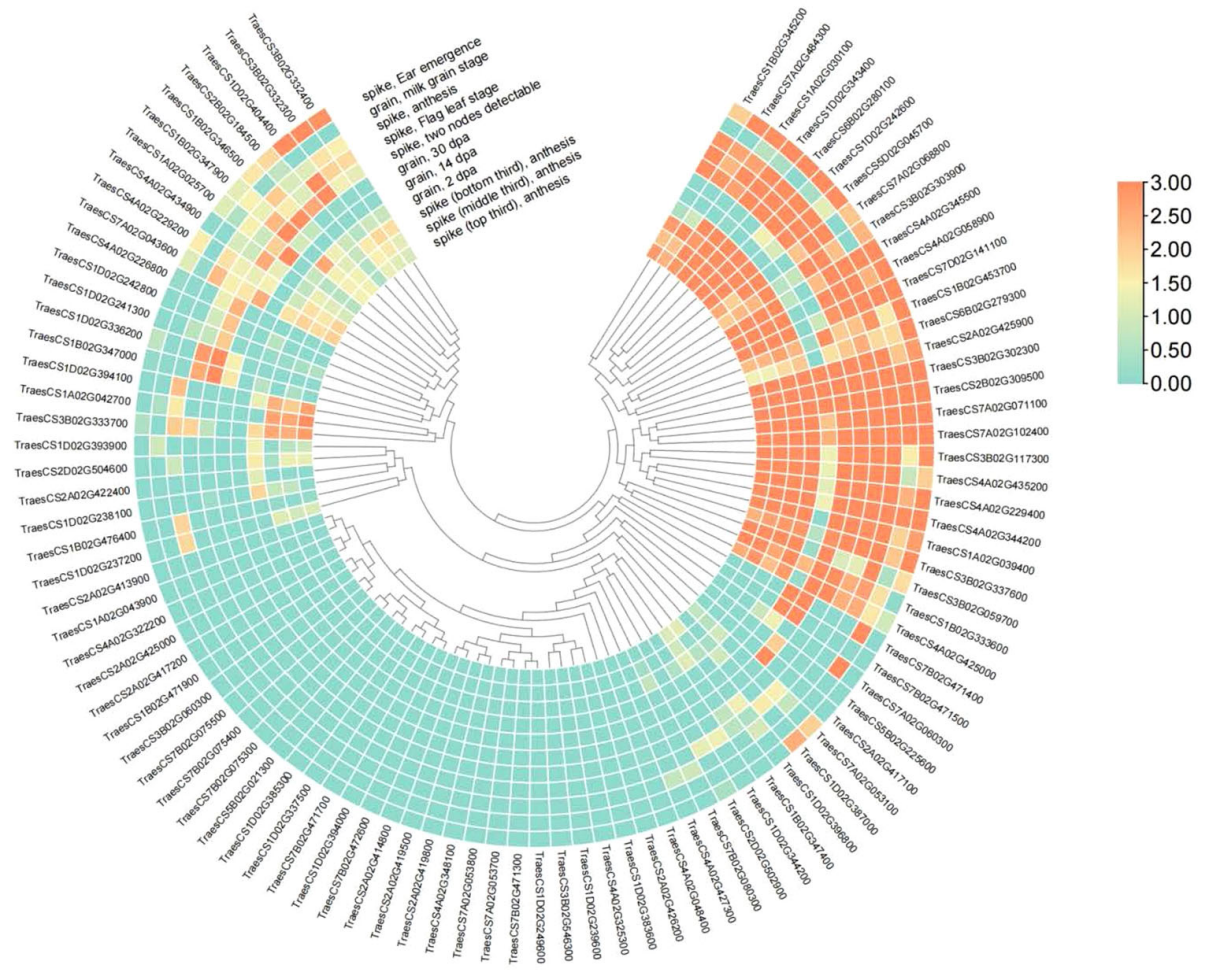
Heatmap showing the differential expression levels of 95 candidate genes.

Upon analyzing the expression of candidate genes, we found that 95 candidate genes had transcripts with TPM > 2 (**Figure 6**; **Supplementary Table S4**). Examining the expression of these genes in spikes and grains, we discovered that *TraesCS3B02G302300*, *TraesCS7A02G071100*, *TraesCS2B02G309500* and other genes exhibited high expression in spikes and grains, suggesting their potential impact on TKW and their candidacy as TKW-related genes (**Figure 6**).

## Discussion

### Identification of key MQTL regions through meta−analysis

MQTL analysis is a potent strategy for validating consistent QTL by integrating independent QTL from different trials onto a consensus or reference map (Goffinet and Gerber, 2000; Saini et al., 2021). Numerous studies on QTL mapping of yield and other significant agronomic traits in wheat have been carried out in recent decades. However, many of the identified QTL in these studies are associated with long CI and low PVE, making them less beneficial for marker-assisted breeding. In contrast, MQTL with narrower CI and relatively high PVE are more convincing in demonstrating their value for molecular breeding (Saini et al., 2022b). Additionally, with continuous advancements in molecular genetics and QTL mapping methods, new QTL are consistently being added to databases. It is highly crucial to stay updated and incorporate these new QTL into more stable and reliable MQTL.

In this study, 993 initial QTL were amassed from 120 studies spanning from 2008 to 2023 to identify genomic regions associated with TKW in wheat. In comparison to subgenomes A and B, the subgenome D had fewer QTL (**Figure 1A**), consistent with previous MQTL analyses for grain yield and other yield-related traits (Liu et al., 2020; Saini et al., 2022b; Soriano et al., 2021; Yang et al., 2021). One potential reason for this observation might be that the subgenome D has a low degree of DNA polymorphism. Unlike the diploid progenitor species *Aegilops* tauschii, extremely low genetic diversity has been detected for the subgenome D of wheat (Mirzaghaderi and Mason, 2019). Concurrently, there is also a restricted gene flow from *Aegilops* tauschii to cultivated wheat (Kumar et al., 2012).

For the 66 MQTL identified in this study, the CI of the identified MQTL, with an average distance of 5.41 cM, was decreased by 3.06-fold in comparison to the mean value of the corresponding initial QTL (**Figure 2**; **Supplementary Table S6**). In a similar study, the discovery of 13 MQTL with an average CI of 13.6 Mb for the initial QTL and 6.01 Mb for the MQTL was found to have a 2.26-fold reduction in contrast to that of the initial QTL for drought tolerance in bread wheat (Kumar et al., 2020). Furthermore, the definite physical position of the 66 MQTL in our study was attained through the publication of the wheat genome reference sequence of Chinese Spring, where the physical position of the identified 66 MQTL ranged from 0.13 Mb (*MQTL.DH.7A.1*) to 52.66 Mb (*MQTL.RIL.5D.2*). Among them, 35 of the identified MQTL contained a 53% genetic CI less than 3 cM (**Supplementary Table S6**).

Twelve core MQTL were selected based on the preferred criteria of at least two overlapping initial QTL with a physical and genetic distance < 1.0 cM (Venske et al., 2019)(**Table 1**). which provided a higher level of confidence for further analysis and for the identification of candidate genes. These twelve core MQTL exhibited a smaller average genetic CI (0.46 cM) compared to that of the initial QTL (16.57 cM), with a 36.03-fold reduction. Among these, *MQTL.DH.1B.3*, *MQTL.DH.2A.1*, *MQTL.DH.2A.3*, *MQTL.DH.3B.2*, and *MQTL.DH.5B.2*, etc., were validated by the MTAs. Regarding the 95 genes obtained through transcriptome and functional annotation, 18 genes were identified within the regions of the twelve core MQTL. Some of the notable characteristics of the twelve core MQTL identified in this study are as follows: They demonstrated stability across various environments. *MQTL.DH.7B.7* consisted of three initial QTL for TKW, showing an average PVE of 13.84% across the DH, F2, and RIL populations, suggesting that *MQTL.DH.7B.7* shows strong stability for the TKW trait. Apart from the above core MQTL, the other core MQTL also showed high stability under different environments. Additionally, most MQTL showed pleiotropic effects.

### Potential candidate genes associated with TKW in meta−QTL regions

To support the location of the MQTL identified in this study, an extensive literature was carried out to identify known genes within MQTL regions. In this study, 18 of the 95 candidate genes homologous to rice genes were found within twelve core MQTL intervals (**Supplementary Table S8**). Among the 6 genes in *MQTL.DH.2A.3*, *TraesCS2A02G417100* and *TraesCS2A02G417200* were homologous to the gene *GW6a* involved in epigenetic mechanisms in rice. *GW6a* encodes a GNAT-like histone acetyltransferase (*Osg1HAT1*). The overexpression of *Osg1HAT1* increases the glume cell number, the grain grouting rate, the grain size and the TKW (Song et al., 2015). *TraesCS2A02G414800*, *TraesCS2A02G419800* and *TraesCS2A02G419500* were homologous to the gene *GW6* involved in GA pathway in rice. Two genes of the GAST family, *OsGASR9* and *GW6*, regulate the grain size and yield and show positive responses to GA treatment (Li et al., 2019; Shi et al., 2020). *TraesCS2A02G413900* was homologous to the gene *OsSPL7/12/16*, *OsmiR535* is highly expressed in young panicles and represses the expression of *OsSPL7/12/16* as well as other downstream genes. *OsmiR535* modulates plant height, the panicle architecture and grain shape possibly by regulating rice *OsSPL* genes (Sun et al., 2019). A key gene, *TraesCS3B02G117300*, in the *MQTL.RIL.3B.5* interval was found to be homologous to the gene *DST* (*WL1*), which controls the grain weight in rice through cytokinin phytohormones. Guo et al. (2020) verified that *OsMAPK6* directly interacts with and phosphorylates *DST* to enhance the activation of *OsCKX2*. The overexpression of *IPT* under drought conditions delays the drought stress responses and increases the production yield (Peleg et al., 2011). A key gene *TraesCS3B02G546300* in the *MQTL.RIL.3B.8* interval was homologous to the gene *CYP96B4* in rice. The *OsmiR396* family members *OsmiR396e* and *OsmiR396f* also reduce GA precursor and *CYP96B4* expression independently to affect the grain size and the plant architecture (Miao et al., 2020). Among the four genes in *MQTL.RIL.7B.3*, *TraesCS7B02G080300* was homologous to the gene *OsOFP8* involved in the BR pathway in rice (Chen et al., 2021). *TraesCS7B02G075300*, *TraesCS7B02G075400*, and *TraesCS7B02G075500* were homologous to the gene *GSA1* (*UGT83A1*) involved in the Auxin pathway in rice. *GSA1* encodes a UDP-glucosyltransferase that regulates the grain size through flavonoid-mediated auxin levels and related gene expression. *GSA1* is also required for the redirection of metabolic flux from lignin biosynthesis to flavonoid biosynthesis, which contributes to the regulation of the grain size and the abiotic stress tolerance (Dong et al., 2020). Three key genes *TraesCS7B02G471300*, *TraesCS7B02G471400*, and *TraesCS7B02G471500* in the *MQTL.RIL.7B.7* interval were homologous to the gene O*sCHT14* involved in ubiquitination and deubiquitination associated with the grain size and the grain weight in rice. *GW2* interacts with chitinase14 (*CHT14*) and phosphoglycerate kinase (*PGK*), both of which are involved in carbohydrate metabolism by modulating their activities or stability (Lee et al., 2018).

Another important finding in the present study was that 4913 putative genes related to TKW were identified within the MQTL regions and exhibited the spatiotemporal and specific expression pattern (**Supplementary Table S9**). In wheat, some genes related to TKW were found in these MQTL regions, such as *APP-A1* within *MQTL.F2.1A.1*, which is associated with wheat particle size (Niu et al., 2023). *TaAGP-L-B1* was found in *MQTL.DH.1B.3*. *TaAGP* is an important rate-limiting enzyme that affects starch synthesis (Kang et al., 2013; Rose et al., 2016; Hou et al., 2017). *TaGS2-2D* was in *MQTL.DH.2D.3*, the plastidic glutamine synthetase isoform (*GS2*) plays a key role in nitrogen assimilation (Li et al., 2011; Hu et al., 2018). *TaCWI-4A* was found in *MQTL.DH.4A.3*, which is related to TKW, heading date, and number of grains per spike (Jiang et al., 2015). *TaSPL14* was found in *MQTL.DH.5B.2*, the *TaSPL14* gene knockout in wheat resulted in a decrease in plant height, spike length, number of spikelet, and TKW, which is similar to the phenotype of *OsSPL14* knockout plants in rice (Cao et al., 2021).

In addition, we annotated 4913 genes using GO or KEGG analysis (**Figures 5**, **6**). KEGG and GO pathway enrichment analysis disclosed that these putative genes were highly involved in the peroxisome, basal transcription factor, tyrosine metabolism, photosynthesis, and plant hormone signal transduction pathways. Peroxisomes are implicated in photorespiration and the synthesis of phytohormones, which are crucial for signaling pathways, including jasmonic acid, auxin, and salicylic acid. Here, a total of 95 putative genes with TPM > 2 in the robust and stable MQTL regions were listed based on significant gene expression in grain that might potentially influence TKW in wheat (**Supplementary Table S9**).

## Conclusion

In this study, we elucidated key genomic regions controlling TKW in 28 DH populations, 16 F_2_ populations, and 76 RIL populations across various environmental conditions in wheat by integrating MQTL analysis and transcriptome assessment. Initially, 242 QTL were identified, which were then refined into 66 MQTL and 12 core MQTL with a mean confidence interval reduction of 36.02-fold compared to the initial QTL. Through genomic sequence comparison, we identified a total of 4913 putative candidate genes within the MQTL regions Moreover, gene expression analysis revealed 95 candidate genes with TPM > 2, indicating high and specific expression levels. We also found 5 overlapping MQTL across diverse genetic populations. Our findings suggest that using diverse genetic populations for TKW QTL mapping can uncover distinct gene enrichment regions, highlighting the importance of considering genetic population diversity in breeding studies. Validation of these key MQTL regions and candidate genes through biological experiments could significantly contribute to the molecular genetic enhancement of TKW in wheat.

## Data Availability

The original contributions presented in the study are included in the article/**Supplementary Material**, Further inquiries can be directed to the corresponding author.
